# The Nurse and Her Advocates

**Published:** 1921-01-08

**Authors:** 


### THE NURSE AND HER ADVOCATES.

It is a singularly unfortunate circumstance that no institution standing in the name of nurses is in a position to make an authoritative statement as to the majority and minority wishes of hospital nurses, or of private nurses. There are some, no doubt, who can guess, and a large number who can voice what in their opinion nurses ought to think. But there is a painful absence of ascertained information which can be analysed and tabulated. This signifies that nursing leaders, commonly so described, have not treated seriously the women whom they have persuaded to join their banners. The College elections have never been conducted according to policies. Candidates are put up because of their personality or prominence, and they are neither informed nor do they inquire what their constituents require or expect of them. And so, when reform is in the air, the nurse is regarded as a machine who neither thinks nor speaks. She is a kind of ward in Chancery, and whatever the Court awards she is expected graciously to accept.

I am afraid that by her negligence she herself contributes to this condition of enslavement. At conferences she is markedly silent, and, instead of discussing her interests, her leaders either talk platitudes or enliven the atmosphere with a little faction fighting.

### A State of Chaos.

The result of all this, in a single word, is chaos. At the moment fevered efforts are being made to do the right thing concerning hours of employment. Nobody holds a mandate, and the person most concerned is inarticulate. For some unexplained reason the Department of Labour is regarded as not sufficiently respectable, and efforts are being made to transfer the functions of Sir Robert Horne to Dr. Addison so far as nurses are concerned. Hours of employment are a matter of supreme and pressing importance. But they are only an item. Another is the training of nurses for Public Health work. In America this has received the most careful attention, and arrangements have been made to ensure that, according to her talents, a probationer may elect to train for service in hospital or for preventive work outside. In England, the career of the nurse has been completely subordinated to circumstances. Hospital authorities have conspired to make her services an involuntary charitable contribution. The three-years' system is designed to suit the hospital rather than to equip the probationer. Any young woman of normal intelligence can learn the art and practice of nursing in two years if she is relieved of the drudgery that ought to be performed by hospital attendants, and her probationary period is made a period of training. This is, of course, heresy. But great truths usually begin life as heresies. I cite these outstanding matters merely as examples. The nurse has a great variety of serious concerns, including pension arrangements, which need careful attention. Nobody is bothering about them. This is my complaint. Pick out any of the prominent so-called "leaders" and ask them what of the night. I gravely doubt if any of them is possessed of a torch. I have been peering round vainly trying to discover a glimmer.

### Forty-eight of Fifty-six?

How is the average nurse to know whether Sir Robert Horne or Dr. Addison is Codlin or Short? The one item that will appeal to her and with force is whether she is to work for forty-eight hours or longer. Where the forty-eight hours' system has been tried it has been found to be a success, and everywhere it has been introduced it was not without previous opposition on the part of hospital governors. It is always the same. Capitalists used to preach against the abolition of child labour, and gravely announce that the country was going to the dogs. The country has not gone to the dogs—and does anybody doubt that if the hospital nurse is granted shorter hours the poor will be any worse off when obliged to go to hospital? I do not blame the hospital governors. They have a very difficult problem to solve, and they deserve great credit for facing it voluntarily and in the public interest. But I do blame the nurses' advocates. I regard their attitude and their dilatoriness as highly discreditable to them. They hold a trust, and they are abusing the confidence of their followers.

In the meantime the nurse is not without her defence. She can find a true and powerful friend in the Press. She can address an audience that could not be contained within the walls of any building and which will include her masters and her colleagues. If her arguments are not altogether sound the weak points will be discovered and the strong ones accentuated. She need have no feeling of shyness about addressing a meeting. In the privacy of her room she can pour forth her soul. In so doing there is nothing to be ashamed of. The nursing profession will degenerate as surely as night follows day if some strong and concerted effort is not made to awaken public opinion to the facts of the situation. Women will decline a service which offers nothing but hardship as a reward for zeal.

IERNE.

